# SUV39H2/KMT1B Inhibits the cardiomyocyte senescence phenotype by down-regulating BTG2/PC3

**DOI:** 10.18632/aging.203551

**Published:** 2021-09-24

**Authors:** Kan Wang, Qiang Zhang Zhu, Xian Tao Ma, Cai Cheng

**Affiliations:** 1 Division of Cardiothoracic and Vascular Surgery, Tongji Hospital, Tongji Medical College, Huazhong University of Science and Technology, Wuhan, Hubei 430030, China; 2 Department of Cardiovascular Surgery, Union Hospital, Tongji Medical College, Huazhong University of Science and Technology, Wuhan, Hubei 430022, China

**Keywords:** cardiomyocyte senescence, oxidative stress damage, SUV39H2, H_2_O_2_

## Abstract

Suppressor of variegation 3-9 homolog 2 (SUV39H2/KMT1B), a member of the SUV39 subfamily of lysine methyltransferases (KMTs), functions as an oncogene in various types of cancers. Here, we demonstrate a novel function of SUV39H2 that drives the cardiomyocyte aging process through BTG2. In our study, cardiomyocyte aging was induced by H2O2 and aging cells exhibited increases in SUV39H2. Knockdown of SUV39H2 accelerated cardiomyocyte senescence, while overexpression of SUV39H2 inhibited the cardiomyocyte senescence phenotype. These effects of SUV39H2 on cardiomyocytes were independent of DNA damage and mitochondrial dysfunction. Interestingly, RNA sequencing and bioinformatics analyses identified a strong correlation between SUV39H2 and BTG2. In addition to this, BTG2 protein levels were significantly increased in SUV39H2-deficient cardiomyocytes, and BTG2 knockdown virtually rescued the cardiomyocyte senescence phenotype induced by SUV39H2 knockdown. Taken together, these results indicate that SUV39H2 protects cardiomyocytes from H2O2 exposure-induced oxidative stress, DNA damage, and mitochondrial dysfunction by regulating the p53-BTG2 pathway. Our findings provide evidence that the activation of SUV39H2 has therapeutic or preventive potential against cardiac aging.

## INTRODUCTION

Cardiac aging, a condition in which heart functional reserve declines, is the primary cause of cardiovascular disease (CVD) [1]. CVD is currently the leading risk factor affecting human life and is also one of the main causes of human mortality [2, 3]. Annually, CVD costs the US healthcare system in excess of $500 billion [4]. It is difficult to improve the cardiac function of older patients because there is no effective therapeutic target to reverse pathological cardiac remodelling [5]. Therefore, we urgently need to fully understand the mechanisms of cardiac aging to improve the quality of geriatric CVD patients [6].

Recently, an increasing number of studies have shown that histone methylation is closely related to aging [7]. Histone methylation is dynamically regulated by methyltransferases and demethylases. Therefore, methyltransferases play a critical role in histone methylation. The suppressor of variegation 3–9 homolog 2 (SUV39H2/KMT1B) is a member of the SUV39 subfamily of lysine methyltransferases (KMTs). In a previous study, SUV39H2 was found to be up-regulated in leukemia on three levels (DNA, mRNA, and protein) [8]. More importantly, Suv39h also regulates telomere length in animals, and Suv39h-deficient mice exhibit gene instability and severely impaired viability [9–11]. As is well known, telomere length is as a hall mark of senescence cells, and SUV39H2 was also shown to regulate telomere length in several types of cells [12, 13]. However, whether SUV39H2 functions in the process of cardiomyocyte aging has not yet been explored.

In our study, we found that SUV39H2 is a highly associated senescence gene expressed in the heart and cardiomyocytes. Knockdown of SUV39H2 can exacerbate cardiomyocyte senescence phenotypes, such as the expression of p21/p53, mitochondrial dysfunction, ROS generation and DNA damage. This process can be blocked by SUV39H2 overexpression. Finally, the results presented herein show that SUV39H2 mediates this process through the p53-BTG2 pathway in cardiomyocytes.

## RESULTS

### SUV39H2 is increased in H9C2 cells treated with H_2_O_2_

**Figure 1:**
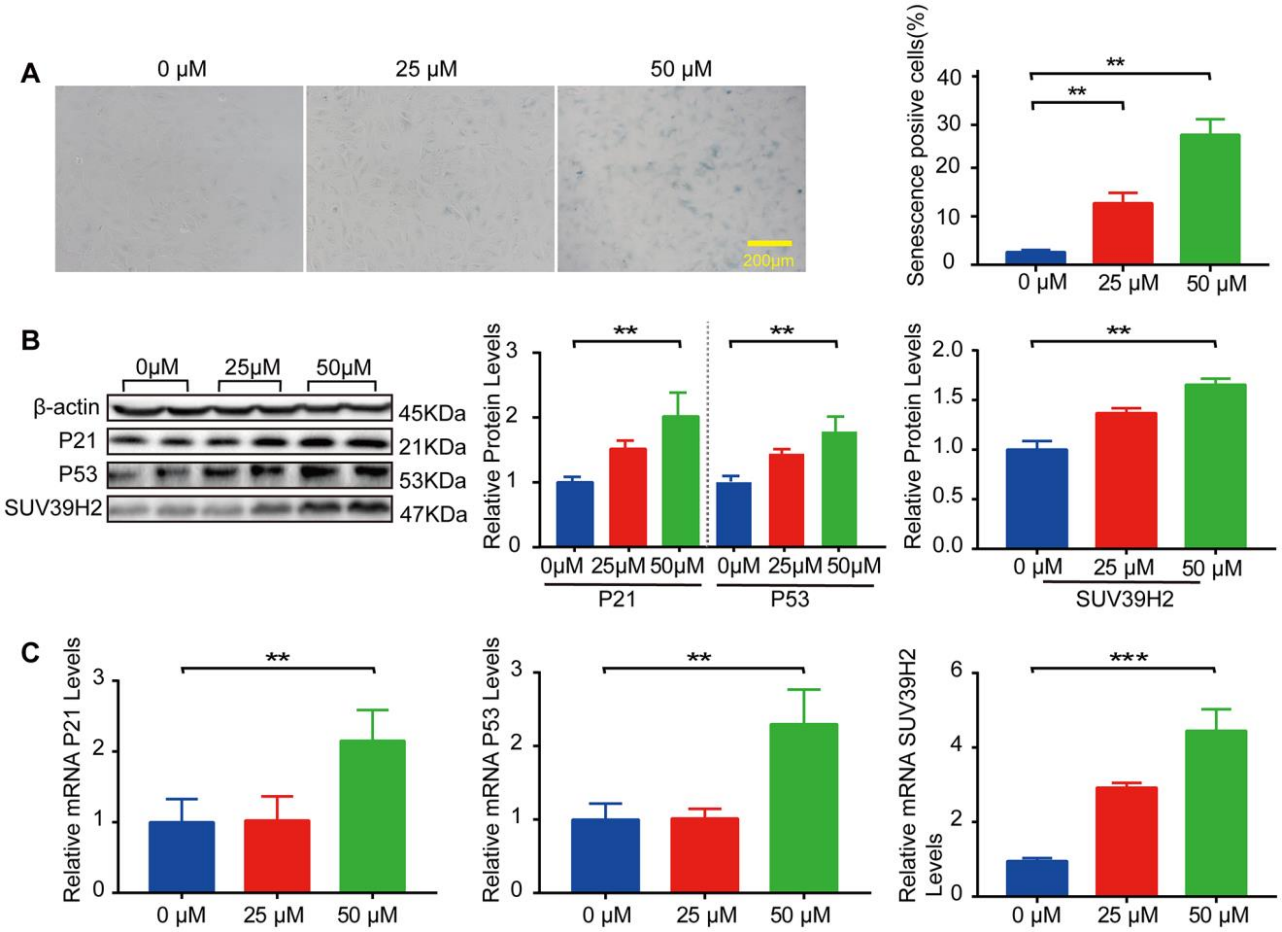
**SUV39H2 is increased in a cardiomyocyte senescence model induced by H_2_O_2_.** (**A**–**C**) Effects of different concentrations of H_2_O_2_ on senescence of H9C2 cells. (**A**) SA-β-Gal staining of H9C2 cells treated with H_2_O_2_ concentration in 0, 25, 50 uM groups. (**B**) Expression of p21, p53, and SUV39H2 in the cells treated with a concentration gradient was detected by western blotting. (**C**) RT-PCR was used to quantify the expression of p21, p53, and SUV39H2 in above groups. All the experiments have been repeated independently at least 3 times. Scale bars, 200 μm. ^*^P < 0.05, ^**^P < 0.01, ^***^P < 0.005 when two groups were compared as indicated, or compared to the corresponding control.

In our study, we used H9C2 cells, a clonal muscle cell line from the rat heart, to perform the experiments. Previous studies have shown that sublethal doses of hydrogen peroxide (H_2_O_2_) induce cellular senescence. To select the appropriate concentration of H_2_O_2_, an SA-β-Gal staining kit was used to detect the degree of cardiomyocyte senescence. H9C2 cells were increased in senescence-positive areas, and the protein and mRNA levels of senescence markers p21 and p53 were elevated after treatment with 50 μM H_2_O_2_ for 48 h (Figure 1A–1C). Subsequently, we examined SUV39H2 expression levels in H9C2 cells after H_2_O_2_ stimulation. The results showed that the mRNA and protein levels of SUV39H2 were significantly elevated in H9C2 cells treated with 50 μM H_2_O_2_ for 48 h compared with the DMEM control (Figure 1B–1C).

### Knockdown of SUV39H2 aggravates senescence in H_2_O_2_-treated H9C2 cells

**Figure 2:**
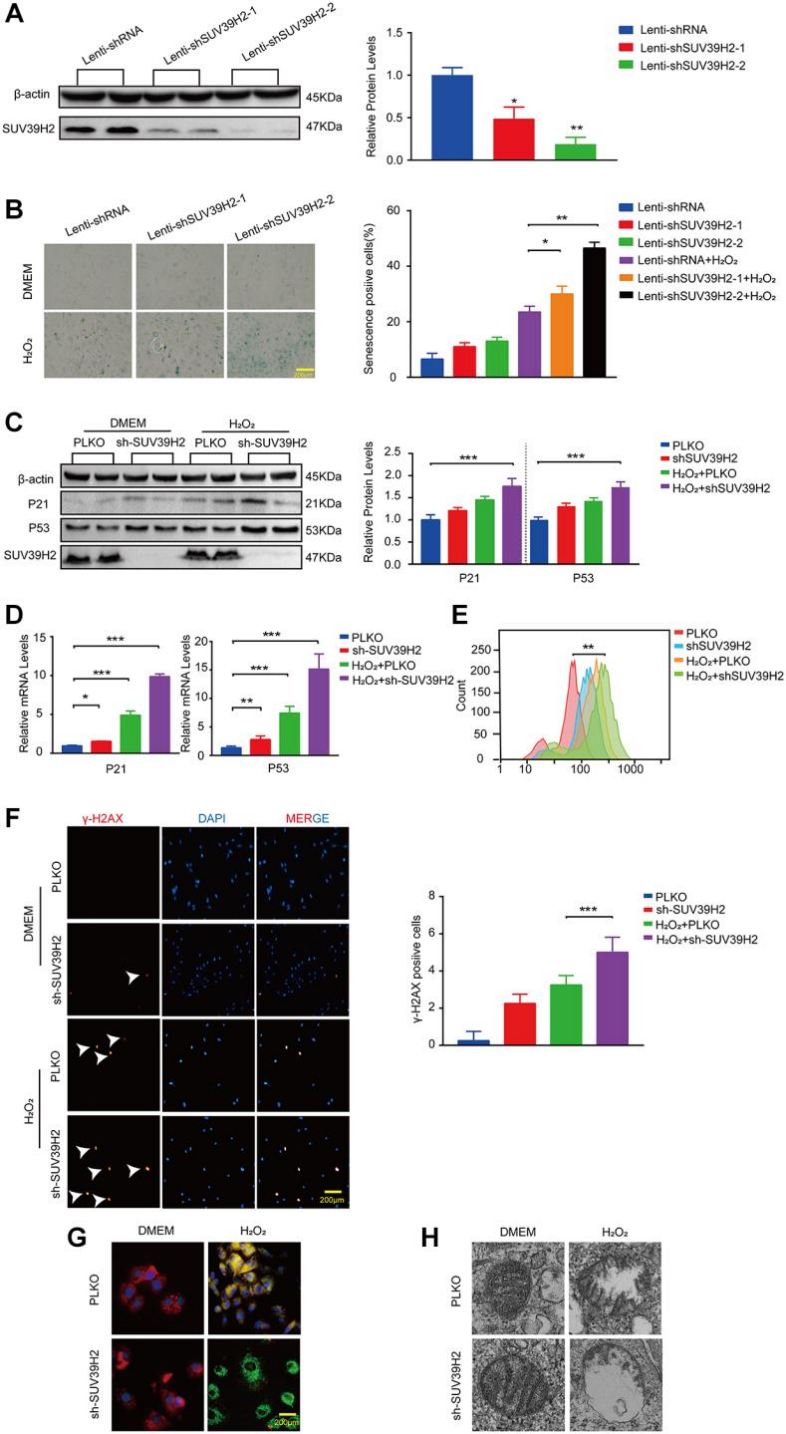
**Knockdown SUV39H2 aggravate senescence in H_2_O_2_-treated H9C2 cells.** (**A**) H9C2 were infected with lenti-shRNA or lenti-shSUV39H2-1 or lenti-SUV39H2-2 in 50 μM H2O2 for 48 hours, then protein levels of SUV39H2 were detected by western blot. (**B**) H9C2 were as described above for 48 hours, and β-galactosidase staining was performed. The positive cells are shown in blue. (**C**–**H**) H9C2 were cultured with basic medium or 50 μM H2O2 supplemented with or without the lenti-sh-SUV39H2 for 48 hours. (**C**–**E**) The protein and mRNA expression of p53, p21, and SUV39H2 were performed by Western blot and qRT-PCR, and ROS generation was detected. (**F**) Detection of γ-H2AX expression in each group by immunofluorescence. (**G**) JC-1 monomer images were shown. The green fluorescence represents JC-1 monomers in 530 nm, whereas red fluorescence represents JC-1 aggregates in 590 nm. (**H**) Electron microscopic images of mitochondrial. All the experiments have been repeated independently at least 3 times. Scale bars, 200 μm.^*^P < 0.05, ^**^P < 0.01, ^***^P < 0.005 when two groups were compared as indicated, or compared to the corresponding control.

The aforementioned results showed that SUV39H2 expression levels were augmented in H9C2 cells upon H_2_O_2_ stimulation. To investigate the correlation between cell senescence and SUV39H2 expression in H_2_O_2_-treated H9C2 cells. We constructed an SUV39H2-knockdown plasmid and created a control cell line, PLKO. We first used western blotting to measure SUV39H2 expression to test the quality of knockdown efficiency (Figure 2A). Unexpectedly, after knockdown of SUV39H2, the percentage (green) and staining intensity of SA-β-Gal-positive cells in the H9C2 cell line increased significantly (Figure 2B). Next, we measured telomerase activity through quantified three telomerase genes (Tert, Terf1, Terf2) levels. It’s necessary for us to know whether mitochondrial dysfunction is involved in cardiomyocyte senescence process. According to the result, we used lenti-sh3-SUV39H2 (hereinafter referred to as lenti-shSUV39H2) for all follow-up experiments. Next, western blotting and RT-PCR was used to detect the expression of p53 and p21 in the H9C2 SUV39H2-knockdown cell line, which was consistent with the results of SA-β-Gal staining (Figure 2C, 2D). Oxidative stress has been reported to be related to the aggravation of cell senescence [14]. Therefore, we used flow cytometry to detect the reactive oxygen species (ROS) concentration in H9C2 cells using 2-dichlorofluorescein diacetate (DCFH-DA) (Figure 2E). Given that ROS derive predominantly from mitochondria, we further used electron to detect the morphology of mitochondria. In addition, immunofluorescence was used to detect the fluorescence intensity of γ-H2AX and JC-1 staining in each group of cells (Figure 2F, 2G). Red fluorescence represents JC-1 aggregate and green fluorescence denotes JC-1 monomer. And electron microscope showed larger and swollen mitochondria in knockdown SUV39H2 H9C2 cells (Figure 2H). As shown in Figure 2G, knockdown SUV39H2 H9C2 cells show relatively significantly higher frequency of mitochondrial depolarization events (green). These results suggest that knockdown of SUV39H2 can cause mitochondrial dysfunction and significantly increase the accumulation of ROS in H9C2 cells, then damage the DNA.

### Overexpression of SUV39H2 alleviate senescence in H_2_O_2_-treated H9C2 cells

**Figure 3:**
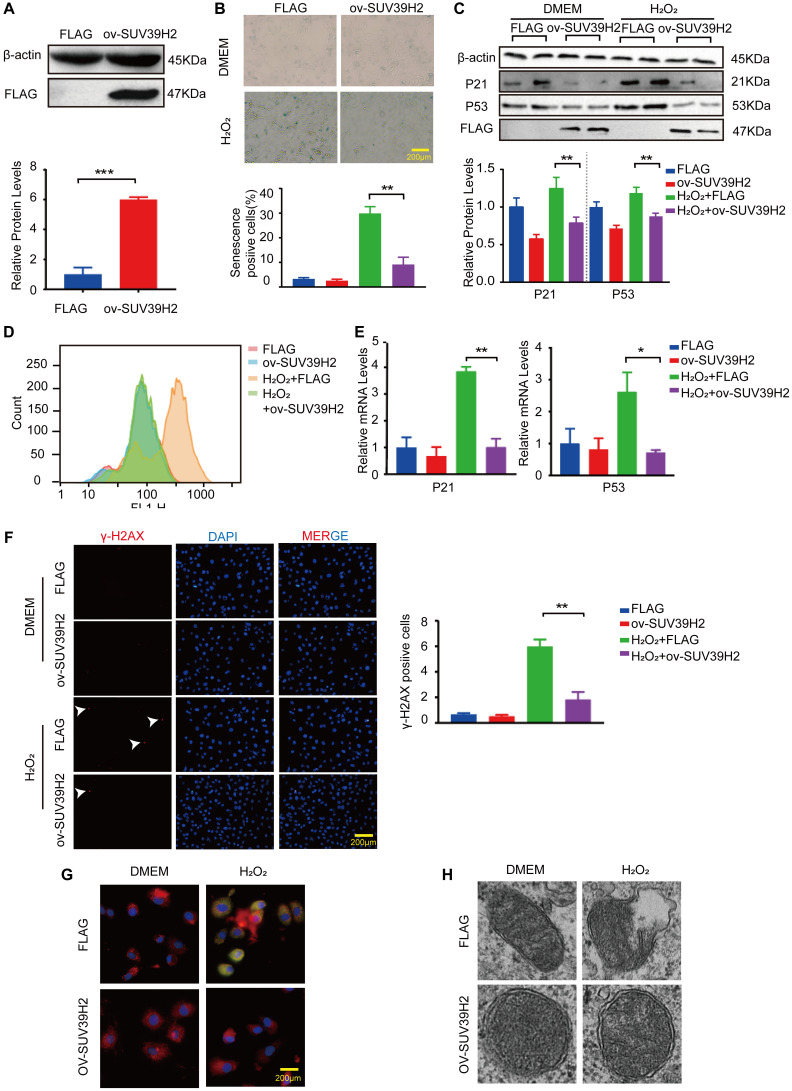
**Overexpression SUV39H2 alleviate senescence in H_2_O_2_-treated H9C2 cells.** (**A**) The SUV39H2-overexpression plasmid was constructed and verified by western blotting. (**B**–**F**) H9C2 were cultured with basic medium or 50 μM H2O2 supplemented with or without the lenti-ov-SUV39H2 for 48 hours. (**B**) The image shows that each group was stained with β-galactosidase, and the percentage of total cells stained with β-galactosidase are blue. (**C**) Western blotting was used to detect the expression of p53, p21 and FLAG. (**D**) Flow detection of ROS yields in each group. (**E**) The expression of p21 and p53 was quantified using RT-PCR. (**F**) Detection of γ-H2AX expression in each group using immunofluorescence. (**G**) Overexpression of SUV39H2 in H9C2 cells stained with JC-1 at 48 hours after treatment with H_2_O_2_. The green dye represents JC-1 monomers in cytoplasm while in red represents JC-1 aggregates in nucleus. (**H**) Electron microscopic images of mitochondrial. All the experiments have been repeated independently at least 3 times. Scale bars, 200 μm. ^*^P < 0.05, ^**^P < 0.01, ^***^P < 0.005 when two groups were compared as indicated, or compared to the corresponding control.

Next, we investigated whether SUV39H2 overexpression had any effect on H9C2 senescence. We constructed a SUV39H2-overexpression plasmid and used lenti-FLAG as a control (negative control) to form a stable H9C2 cell line. As shown in Figure 3A, SUV39H2 expression was prominently increased in H9C2 cells infected with lenti-SUV39H2. The results of SA-β-Gal staining also showed that overexpression of SUV39H2 significantly reduced the number of senescent-positive cells (Figure 3B). When compared with the lenti-FLAG group, p53 and p21 levels were also significantly decreased after SUV39H2 overexpression. (Figure 3C). Consistent with the above results, we evaluated the levels of oxidative stress, DNA damage, and mitochondrial function in H9C2 SUV39H2-overexpressing cell lines. The results showed that compared with H9C2 cells in the control group, the level of ROS in SUV39H2-overexpressing cell lines decreased (Figure 3D, 3E). In addition, the immunofluorescence results also showed that the expression of γ-H2AX SUV39H2-overexpressing cell lines was down-regulated compared to the control group (Figure 3F). Moreover, this stable cell lines shows no obvious JC-1 monomer signal (green) and damage in mitochondrial morphology (Figure 3G, 3H).

### RNA sequencing of H9C2 cells with SUV39H2-knockdown induced by H_2_O_2_

**Figure 4:**
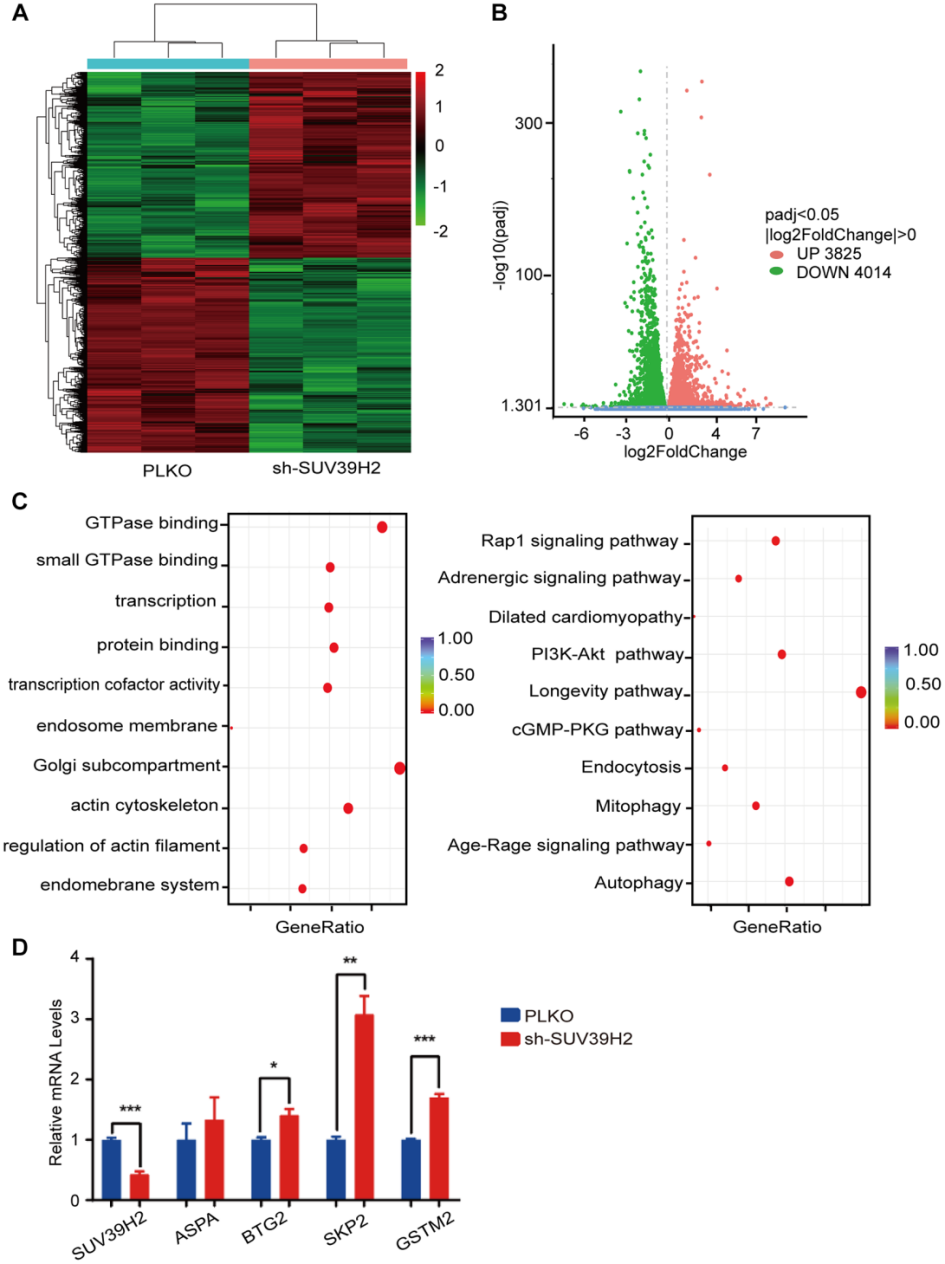
**RNA-seq of knockdown SUV39H2 in cardiomyocytes induced by H_2_O_2_.** (**A**–**D**) Following 50 μM H_2_O_2_ treatment of the PLKO and sh-SUV39H2 groups, RNA-Seq data processing. (**A**) Heat map showing the differential gene expression between the two groups. (**B**) Volcano map showing that there were 3,825 up-regulated genes and 4,014 down-regulated genes between the two groups. (**C**) Bubble chart showing the top 10 enrichment paths from GO and KEGG analyses. (**D**) Quantification of the differential genes after RNA-Seq analyses of the cells of the two groups. ^*^P < 0.05, ^**^P < 0.01, ^***^P < 0.005 when two groups were compared as indicated, or compared to the corresponding control.

To investigate the inherent anti-aging mechanism of SUV39H2, we used RNA sequencing (RNA-seq) to analyze the gene expression profile of H9C2 SUV39H2-knockdown cell lines treated with H_2_O_2_. Compared with the control group, the results showed that 7,839 genes were differentially expressed in the shSUV39H2 group, of which 3,825 genes were up-regulated and 4,014 down-regulated (Figure 4A–4B). We utilized gene ontology (GO) annotation analysis to help identify differentially expressed genes targeted by SUV39H2. The results showed that most of the differentially expressed genes were related to biological processes rather than molecular functions and cellular components. Kyoto Encyclopedia of Genes and Genomes (KEGG) pathway analysis showed that the differentially expressed genes were enriched for the longevity-regulating pathway, cGMP-PKG signal pathway, PI3K-Akt signal pathway, Rap1 signal pathway, and autophagy-related pathways (the 10 pathways with the highest enrichment scores) (Figure 4C). According to the results of GO and KEGG analyses, we found that a large number of differentially expressed genes were mainly related to cell growth and proliferation-related pathways, which may be the key to cardiomyocyte senescence induced by H_2_O_2_. Quantitative real time PCR (qPCR) was used to confirm the initial results obtained by RNA-seq, and we found four up-regulated genes, including SUV39H2, which is consistent with our sequencing results (Figure 4D). And this results can be repeated by another shRNA (sh2-2-SUV39H2) (Supplementary Figure 1A).

### BTG2 plays a role in regulating SUV39H2-knockdown cardiomyocyte senescence induced by H_2_O_2_

**Figure 5:**
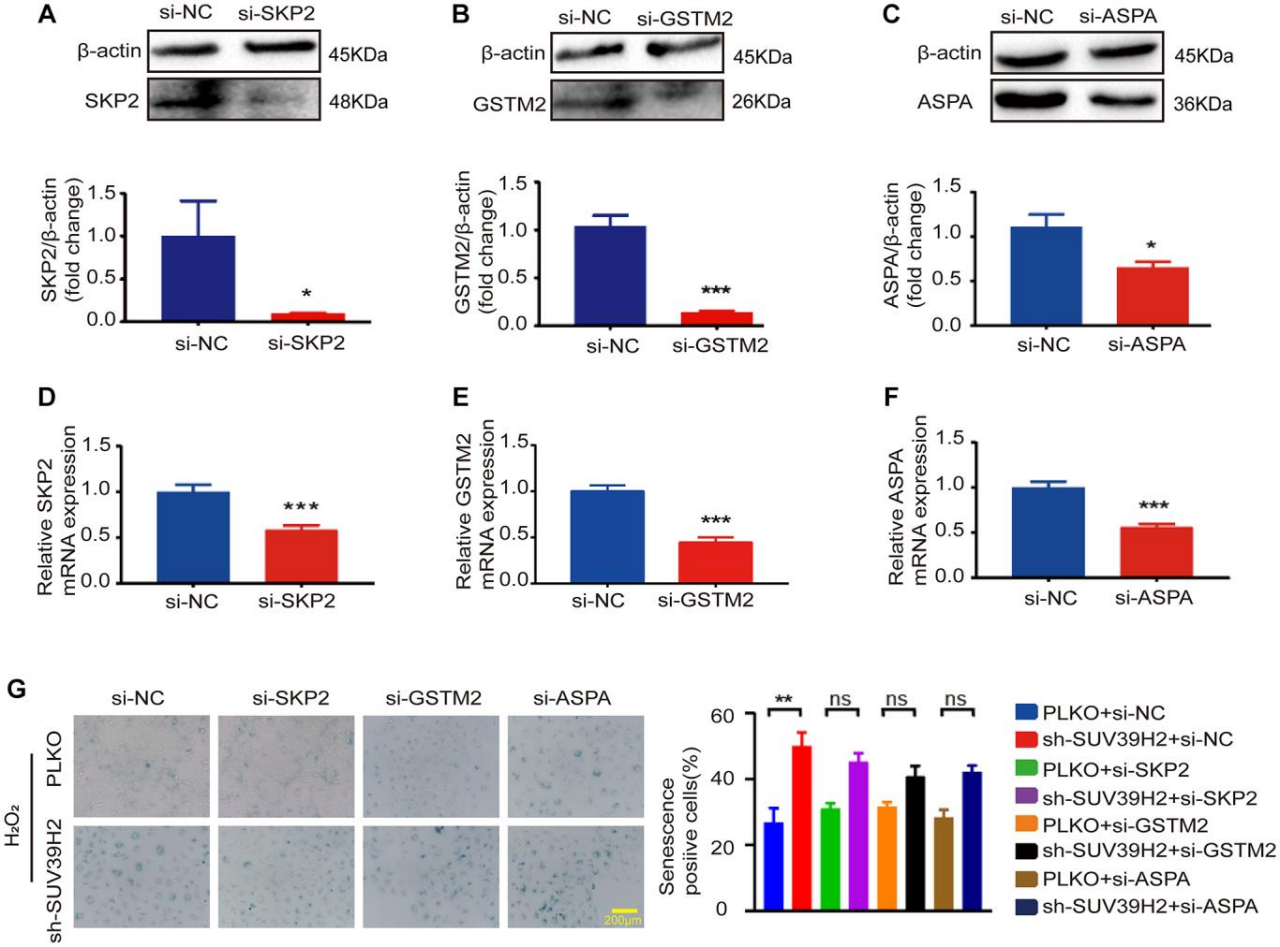
**SKP2, GSTM2, and ASPA silencing did not affect the level of sh-SUV39H2-induced cardiomyocyte senescence.** H9C2 cells were pretreated with 5% DMEM for 8 h, and then transfected with interfering RNA and control si-NC for 48 h. Transfection efficiency was analyzed and quantified by western blotting and RT-PCR. There are three siRNA sequence: SKP2 (**A**, **D**), GSTM2 (**B**, **E**), GSTM2 (**C**, **F**). The blue region shows senescence-positive cells, and the proportion of positive cells in three random fields was quantified. No differences were observed across experiment groups (**G**). Data are expressed as the mean ± standard deviation. All the experiments have been repeated independently at least 3 times. ^*^P < 0.05, ^**^P < 0.01, ^***^P < 0.005 when two groups were compared as indicated, or compared to the corresponding control.

**Figure 6:**
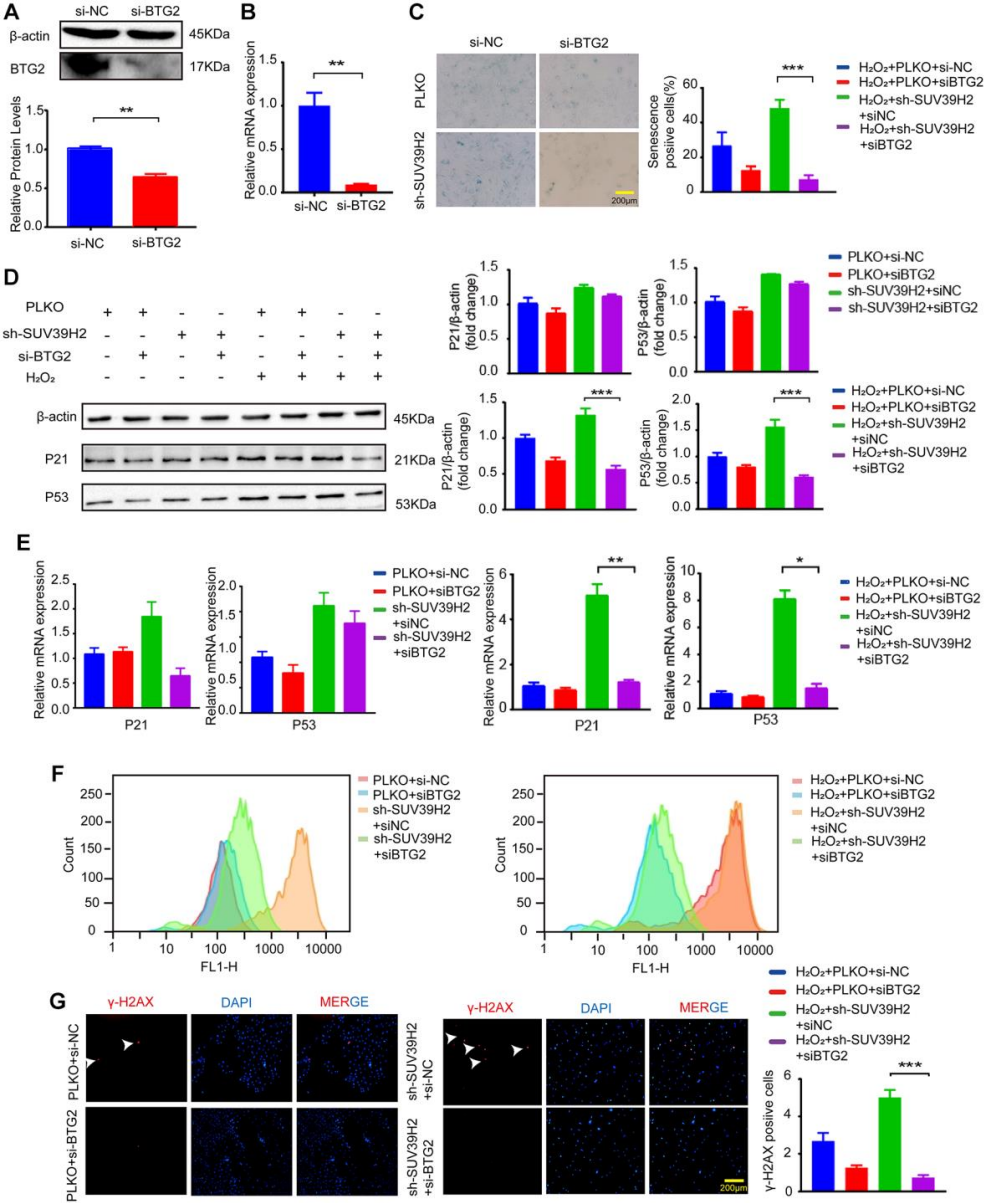
**BTG2 reversed the aging phenotype in knockdown SUV39H2 H9C2 cells.** (**A**–**B**) After transfection of si-BTG2 and control si-NC into H9C2 cells, the cells were collected 48 h later for RT-PCR and western blotting. (**C**) PLKO-H9C2 and sh-SUV39H2-H9C2 cell lines were cultured in 50 μM H_2_O_2_ added with or without siBTG2 for 48 hours. And an SA-β-Gal staining kit was used to detect senescence cells. (**D**–**G**) PLKO-H9C2 and sh-SUV39H2-H9C2 cell lines were cultured in normal medium or 50 μM H_2_O_2_ medium added with or without siBTG2 for 48 hours. (**D**) Western blotting for p21 and p53 in mentioned above groups were performed, and the level of β-actin protein was measured as the control. (**E**) The expression of p21 and p53 in mentioned above groups were quantified by RT-PCR. (**F**) Flow cytometry was used to quantitatively detect the ROS production of mentioned above groups. (**G**) The cells in mentioned above groups were fixed and analyzed by immunofluorescence to detect the expression level of γ-H2AX. All the experiments have been repeated independently at least 3 times. ^*^P < 0.05, ^**^P < 0.01, ^***^P < 0.005 when two groups were compared as indicated, or were compared to the corresponding control.

Based on the above results, to determine the downstream genes of SUV39H2, we constructed siRNA for four selected genes and used si-NC (negative control) as a control. RT-PCR was used to test up-regulated genes’ siRNA knockdown efficiency, and the knockdown efficiency of siBTG2-1 and siBTG2-3 was not satisfactory (Supplementary Figure 1B, Figure 5A–5F, Figure 6A–6B). Therefore, we used siBTG2-2 (hereinafter referred to as siBTG2) for all follow-up experiments. We then examined the impact of the four siRNAs on the knockdown of SUV39H2 in H9C2 cells when treated with H_2_O_2_. Our results showed that only BTG2 knockdown largely rescued H9C2 cells from cell senescence induced by SUV39H2 knockdown (Figure 5G, Figure 6C–6E). The fluorescence intensity of ROS and γ-H2AX also showed that si-BTG2 induced low oxidative stress and low DNA damage in H9C2 cells (Figure 6F–6G). Overall, these results indicate that SUV39H2 inhibits cell senescence by regulating BTG2 expression.

## DISCUSSION

**Figure 7:**
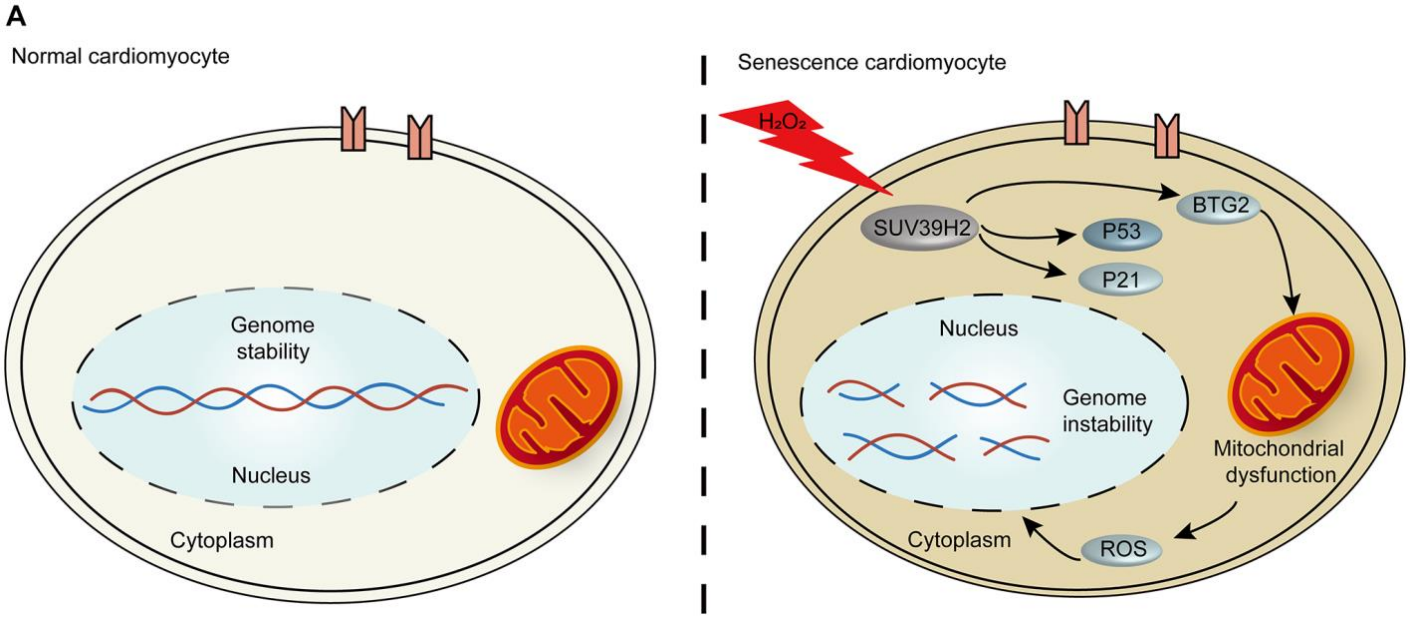
**The p53-BTG2 pathways play a role in regulating senescence in SUV39H2-knockdown cardiomyocytes induced by H_2_O_2_.** (**A**) Scheme indicating the proposed mechanism of senescence regulation by SUV39H2 in H9C2 cells.

Aging is the greatest risk factor for CVD. Recent studies have indicated that the pathogenesis of cardiac aging is directly related to heart diseases, such as heart failure [15]. Patients with a CVD such as heart failure have a lower quality of life and high mortality rates [16]. Due to the non-renewability of cardiomyocyte cells, the vast majority of patients with aging-related CVD cannot escape disease recurrence [17]. Therefore, further investigation into the mechanism behind cardiomyocyte cell senescence and strategies to inhibit the development of cardiomyocyte senescence will be beneficial for patients’ quality of life. Our results suggest that the oncogene SUV39H2 decreases the level of cardiomyocyte cell senescence through the p53-BTG2 pathway (Figure 7A).

A previous study reported that myocardial aging was accidentally identified in a heart disease model in which chemotherapeutic drugs were used [18]. This kind of cell senescence caused by drugs or exogenous factors was termed “stress senescence” in later studies. Our study established a cell senescence model with 50 μM H_2_O_2_ and found that SUV39H2 (a type of lysine methyltransferase) was increased (Figure 1B–1C). How does SUV39H2 regulate the aging of cardiomyocytes? A previous study showed that overexpression of SUV39H2 contributed to the progression of cancer [8, 19–21], and this phenomenon is the opposite of the aging process. Recent studies also have shown that the SUV39H2-deficient mouse exhibits growth inhibition and SUV39H2 is strongly related to the regulation of telomere length associated with aging [10–12]. As expected, knockdown of SUV39H2 promoted H_2_O_2_-induced senescence, as shown by SA-β-Gal staining, western blotting, the level of γ-h2ax expression, ROS generation, and mitochondrial membrane potential (Figure 2B–2F). In addition, our results were consistent with a previous study that also showed that oncogene or stress-induced senescence does not rely on telomere shortening (Supplementary Figure 1C) [22]. Moreover, SUV39H2 knockdown causes cells, such as HeLa cells and RERF-LC-AI cells, to become more sensitive to stress [23], corroborating our previous results. Meanwhile, SUV39H2 knockdown also increased ROS levels, the strength of which was shown by the DCFH-DA assay (Figure 2D). In addition, our results showed that knockdown of SUV39H2 alone also has similar effects, which are lower than those of the experiments combined. Initially, we considered these factors to be synergistic, but after overexpression of SUV39H2 in H9C2 cells induced by H_2_O_2_, we found that the effect of H_2_O_2_ was occluded by overexpression of SUV39H2. This result indicates that SUV39H2 has a strong direct relationship with the effects of H_2_O_2_. To investigate this hypothesis further, we decided that other cell lines should be examined. Therefore, we chose five types of human cell lines and treated them with lentiviruses to induce knockdown and overexpression. We have added six cell lines to illustrate this important question. These six cell lines are HEPG2 (Human Hepatic cells), A549 (Human lung adenocarcinoma cells), SNU398 (Human Hepatic cells), SK-CO-1 (Human colorectal adenocarcinoma cells) and Hela (Human Cervical cancer cells). As shown in Supplementary Figure 1D and 1E. The result showed that critical experiments could be repeated in human cancer cell lines.

After identifying SUV39H2 as a potential therapeutic target, we successfully found that overexpression of SUV39H2 attenuated cell senescence markers, such as γ-H2AX, SA-β-Gal activity, p21/p53, and ROS (Figure 3B–3F). Next, by analyzing enriched GO terms and KEGG pathways through RNA-Seq results, we discovered that SUV39H2 might reverse these changes by altering the longevity-regulation pathways associated with cell progression and growth (Figure 4C). The increases in BTG2 with the H_2_O_2_-induced knockdown of SUV39H2 in H9C2 cells aroused our attention.

BTG2/PC3 has been shown to stimulate senescence through regulation by p53 [24]. Another study showed that the expression of BTG2 regulates the G1/M cell cycle transition by promoting the expression of CDK4 [25, 26]. To examine the influence of BTG2 on the cell senescence of SUV39H2-knockdown cardiomyocytes, we showed that knockdown of BTG2 by siRNA reversed the senescence aggravated by knockdown of SUV39H2 in H_2_O_2_-treated H9C2 cells (Figure 6C–6G). Thus, we consider BTG2 a master regulator downstream of SUV39H2 in the process of cardiomyocyte senescence (Figure 7A).

In this study, our data demonstrate the function of SUV39H2 in H9C2 cells, and we found that SUV39H2 knockdown induces mitochondrial dysfunction and DNA damage in cardiomyocytes. Finally, our study showed that SUV39H2 can down-regulate p53 to decrease the expression of BTG2 and alleviate senescence of H9C2 cells induced by H_2_O_2_. These observations indicate that SUV39H2 has a potential role in cardiac senescence and may be a novel therapeutic target for pathological cardiac senescence.

## MATERIALS AND METHODS

### Cell culture and treatment

The H9C2 cell line was obtained from the American Type Culture Collection (USA). H9C2 cells were grown in DMEM containing 10% fetal bovine serum (FBS; Gibco-BRL, Australia) supplemented with 1% penicillin antibiotics. According to the approved culture protocols for this cell line, H9C2 cells were cultured in a humidified incubator with 5% CO_2_ at 37°C. Cells were treated with 0, 25, and 50 μM H_2_O_2_ (Sigma-Aldrich, USA) and incubated for 48 h.

### Plasmid construction

Positive and negative primers were obtained after designing shRNA sequences using the BLOCK-iT biology RNAi Designer (Thermo Fisher, USA). The sequences of primers used (Shanghai Bioengineering Co., Ltd., China) are shown in Table 1. The plasmids with two restriction sites EcoRI and AgeI, were mixed with positive and negative primers and Ligation High (#LGK-101; TOYOBO, Japan) to ligate overnight, to obtain complete plasmids. Plasmid construction was verified by sequencing and western blotting.

**Table 1:** Primer sequences used for quantitative real-time PCR (RT-PCR).

**Primer**	**Forward Sequences (5′–3′)**	**Reverse Sequences (5′–3′)**
**P21**	AGTATGCCGTCGTCTGTTCG	GAGTGCAAGACAGCGACAAG
**P53**	CCCATCCTTACCATCATCACG	CAGGCACAAACACGAACCT
**SUV39H2**	TCGGCTTCCCAGGATAGCAT	TAACCTCTGCACGTCTCAGC
**18s**	TACCACATCCAAGGAACAGCA	TGGAATTACCGCGGCTGCTGGCA
**Skp2**	GTGCCTCCCTGAGCTTTTGAG	GGTGCAGATTTTTGCCTGCG
**Btg2**	CGCACTGACCGATCATTACAAA	GATGCGGTAGGACACTTCGT
**ASPA**	ACTGGCTAAAGAATGGCGCT	GGGAAGGATGCTCGATGAGG
**Gstm2**	AAGCACAACCTTTGTGGGGA	CTTGCCCAGGAATTCGGAGT

### Lentiviral infection of H9C2 cells

H9C2 cells were infected with the viral solution produced by co-transfection of 293T cells with PMD2G and SPEX2 (2:1:1). H9C2 cells were infected with the lentivirus for 24 h; they were selected using 2 μg/mL puromycin for 2 d, 48 h later. H9C2 cell lines with SUV39H2 stably knocked down or over-expressed were obtained.

### Cell transfection

Targeted knockdown BTG2/SKP2/ASPA/GSTM2 was generated using short interfering RNA (siRNA) (Ribbio, China). Lipofectamine 3000 reagent (Invitrogen, USA), was used to transfect the siRNA into H9C2 cells, and the cells were cultured in the transfection mix for 3 days according to the manufacturer’s instructions. The target sequences of the siRNAs are shown in Table 2.

**Table 2:** Oligo sequences used for siRNA.

**siRNA**	**Target Sequences (5′–3′)**
**BTG2**	GCAGAGACTCAAGGTTTTC
**SKP2**	GCAGATTAATTGTGCCTAT
**GSTM2**	CCTTGATCAACACCGTATA
**ASPA**	CTCGTTCCATTGCCAAGTA
**Negative control**	UUCUCCGAACGUGUCACGUTT

### ROS assay

The cells were covered with a serum-free medium containing 2B7-dichlorofluorescein diacetate (DCFH-DA) (Beyotime Biotechnology, China) and cultured in a 37°C incubator in the dark for 20 min. After the cells were washed three times, they were digested and centrifuged for collection. After resuspension in PBS, the level of ROS production was determined by flow cytometry. FlowJo software (Tree Star, USA) was used to analyze the ROS peak values.

### Immunofluorescence

The fluorescence of cells was observed in a dark room using a fluorescence microscope. The treated cells were fixed in 4% paraformaldehyde for at least 20 min, washed three times using PBS, permeabilized using 0.2% Triton for 15 min, followed by washing three times using PBS, then sealed at 4°C with 5% goat serum (diluted by PBS) and incubated overnight with an anti-γ-H2AX antibody (ab26350; Abcam, USA). Under dark conditions, the cells were incubated with secondary antibodies for 2 h (diluted 1:200). The cells were covered with 6-diamidino-2-phenylindole (DAPI) (Beyotime) for 10 min for nuclear staining, and then immediately observed under an Olympus fluorescence microscope (Olympus America Inc., USA).

### SA-β-gal staining

The 6-well plate cells were washed using PBS and stained with the SA-β-gal staining kit (Cell Signaling, USA). In a nutshell, the cells were fixed with 1× Fixative Solution for 15 min, then the β-gal working solution (930 μL 1× Staining Solution, 10 μL 100× Solution A, 10 μL 100× Solution B, and 50 μL 20 mg/ml X-gal stock solution) was added and they were placed in a carbon dioxide-free incubator at 37°C overnight. The area of blue cells was then observed under an inverted microscope (200× total magnification). We observed and recorded the proportion of positive blue cells in each group of three visual fields in three separate experiments.

### mRNA extraction and RT-PCR

After completion of the treatment, the total RNA extracted by TRIzol reagent was inversely converted to 3 μg cDNA using the reverse transcription kit Hiscript III-RT SuperMix for qPCR, according to the manufacturer’s instructions (Vazyme, Japan). The primers listed in Table 2 were used for the PCR amplification. The primers (Shanghai Bioengineering, China), cDNA, ChamQ Universal SYBR qPCR Master Mix, and non-RNase ddH_2_O were thoroughly mixed according to the kit instructions, and then added to the 96-well PCR plate (Bio-Rad, USA), and placed into a real-time PCR detection system (Bio-Rad). The data were derived as multiple relative changes (2^−ΔΔCT^) and standardized according to expression of the control gene (GAPDH/18S). The experiment was repeated at least three times, and the results were expressed as mean ± standard deviation (SD).

### Western blotting

Briefly, the treated cells were fully cleaved using RIPA buffer (Beyotime Biotechnology), lysed with the assistance of ultrasound, and centrifuged at 12,000 g at 4°C for 15 min. The supernatant was denatured at 95°C after adding 5× SDS running buffer, and total protein was obtained. The total protein was electrophoresed on a 12% sodium dodecyl sulfate-polyacrylamide gel and transferred to a polyvinylidene fluoride (PVD; 0.22 μm) membrane (Millipore Co., USA). The PVDF membrane was then sealed with 5% skimmed milk prepared in TBST solution, and then incubated overnight with anti-P21 (A2691; ABclonal, China), anti-p53 (A1803, ABclonal), anti-SUV39H2 (190870, Abcam), anti-BTG2 (CAB9848; Antibody Genie, USA), anti-GSTM2 (175282, Abcam), anti-ASPA (223269, Abcam), and anti-SKP2 (183039, Abcam) at 4°C. The membrane was then incubated with the secondary antibody (goat anti-rabbit antibody, ab205718, Abcam) for 1 h, and then washed twice using TBST. The Super Signal™ West Pico PLUS chemiluminescence substrate was used to detect proteins on the PVDF membrane (Bio-Rad). Image Lab (Bio-Rad) was used to view and analyze the images of protein imprinting.

### RNA-Seq

After the cells were prepared, sequencing and analyses were performed by the Beijing Novo Gene Company (USA). In brief, total RNA was extracted from H9C2 cells using TRIzol reagent. After quantitative and qualitative analyses, 1 μg was used as the input material for each group of samples. According to the manufacturer's instructions, TruSeq PE Cluster Kitv3-Cbot-HS (Illumina, USA) was used to prepare the RNA library, and the Agilent Bioanalyzer 2100 system (Agilent, USA) was used to evaluate the quality of the library. The library was sequenced on an Illumina NovaSeq platform. Differential expression analyses of two conditions/groups (two biological replicates per condition) were performed using the DESeq2 R package (ver. 1.20.0). Genes with an adjusted *P*-value < 0.05, as identified by DESeq2, were assigned as differentially expressed.

### GO and KEGG analyses

The GO and KEGG enrichment analysis of the differential genes identified by sequencing was carried out by using the modified gene length cluster Profiler R software package. GO and KEGG items with *P* < 0.05 were considered to be significantly enriched for differentially expressed genes. KEGG is a database resource for understanding high-level biological functions and utilities, such as those of the cell, the organism, and the ecosystem, derived from molecular-level information (http://www.genome.jp/kegg/). We identified the top 10 enriched genes of the differentially expressed genes obtained from GO and KEGG analyses.

### JC-1 detection

JC-1 was used to valuate mitochondrial membrane potential detection. Before the cells to be processed, plant the glass plate into the well plate and wait for the cell climbing film. After removing the original medium from the treated cells, wash the cells once with PBS. Add 1ml of JC-1 staining working solution, mix well and incubate in a 37°C incubator for 20 minutes. After the incubation, discard the supernatant and wash twice with JC-1 staining buffer. Pick out the cell slide and place it under a laser confocal microscope for observation.

### Electron microscopy

Electron microscopy was used to evaluate the morphology of mitochondrial. Rinse the treated cells with PBS and add a sufficient amount of 4°C pre-cooled special fixative for electron microscope to fix for 1 hour, then use a cell scraper to scrape the cells and transfer them to a 15 ml centrifuge tube. Drop the sample on the special copper net for 3–5 minutes. Drop 2% phosphotungstic acid on the copper net and leave it for 2–3 minutes. Observation by electron microscope (HT7700 Bio-TEM, Japan).

### Statistical analysis

The data were analyzed using GraphPad Prism ver. 7.0 (Graph Pad Software, USA). Comparisons between the data groups were carried out using an unpaired sample *t*-test, and the results were expressed as the mean ± SD. Each experiment was divided into three parallel groups, and the experiment was repeated three times, independently and randomly. A *P*-value was less than 0.05 was taken to indicate a statistical difference between the two groups of data.

## Supplementary Materials

Supplementary Figure 1
