# Supplementary material for: SUV39H2/KMT1B Inhibits the cardiomyocyte senescence phenotype by down-regulating BTG2/PC3
Source: Aging (Albany NY). 2021 Sep 24;13(18):22444–58. doi: 10.18632/aging.203551 (PMC8507256; doi:10.18632/aging.203551)
Supplement: Supplementary Figure 1 [file aging-13-203551-s001.pdf]

SUPPLEMENTARY FIGURE

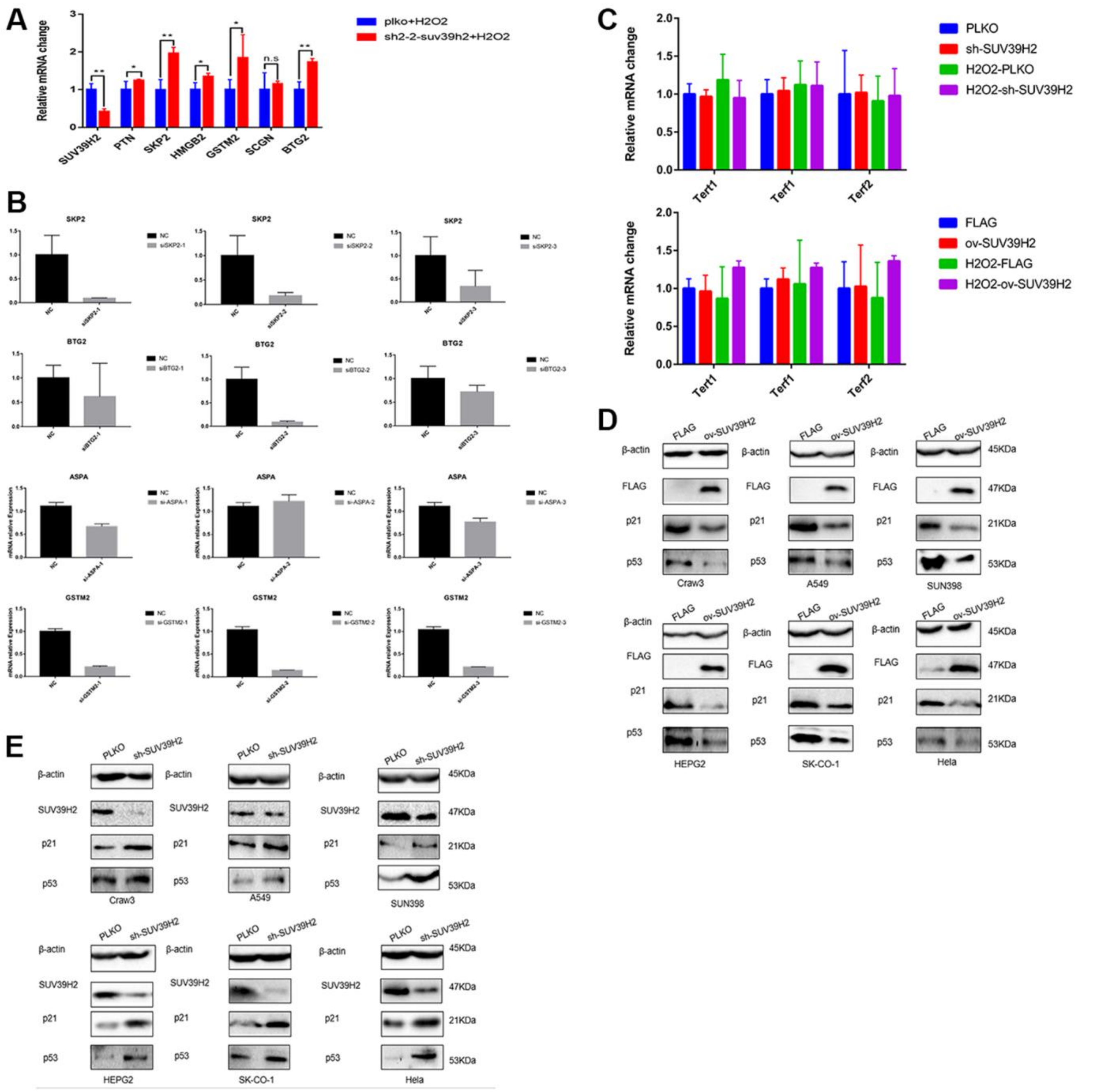

Supplementary Figure 1. (**A**) Another shRNA (sh2-2-SUV39H2) was used to examine the result of RNA-seq through. (**B**) qPCR was used to evaluate knockdown efficiency in three parallel siRNA (SKP2/BTG2/GSTM2/ASPA). (**C**) PLKO-H9C2, sh-SUV39H2-H9C2, FLAG-H9C2 and ov-SUV39H2-H9C2 cell lines were added with or without 50 μM H_2O_2 for 48 hours, and telomerase activity was detected by qPCR using telomerase associated genes (Tert1, Terf1, Terf2). (**D**–**E**) HEPG2 (Human Hepatic cells), A549 (Human lung adenocarcinoma cells), SNU398 (Human Hepatic cells), SK-CO-1 (Human colorectal adenocarcinoma cells) and Hela (Human Cervical cancer cells) were transfected with control, lenti-sh-SUV39H2 and lenti-ov-SUV39H2 in 50 μM H_2O_2. Western blotting for p21 and p53 in mentioned above groups were performed. All the experiments have been repeated independently at least 3 times. $^{*}P < 0.05$, $^{**}P < 0.01$, $^{***}P < 0.005$ when two groups were compared as indicated, or were compared to the corresponding control.
